# Synergistic Effect of *Rubus crataegifolius* and *Ulmus macrocarpa* Against *Helicobacter pylori* Clinical Isolates and Gastritis

**DOI:** 10.3389/fphar.2020.00004

**Published:** 2020-02-20

**Authors:** Jung Uoon Park, Jin Sook Cho, Jong Seok Kim, Hyun Kyu Kim, Young Hee Jo, Md Aziz Abdur Rahman, Young Ik Lee

**Affiliations:** ^1^ Industrial Bioresource Research Center, Korea Research Institute of Bioscience and Biotechnology (KRIBB), Daejeon, South Korea; ^2^ Lee's Biotech Co., KRIBB, Daejeon, South Korea; ^3^ College of Medicine, Myunggok Medical Research Institute, Konyang University, Daejeon, South Korea; ^4^ R & D, Kolmar BNH Co., Ltd., Sejong, South Korea

**Keywords:** *Rubus crataegifolius*, *Ulmus macrocarpa*, MIC, *Helicobacter pylori*, gastritis

## Abstract

*Helicobacter pylori* is one of the most widespread infections involved in the pathogenesis of chronic gastritis, peptic ulcer, and gastric cancer. Hence, there is an urgent need to develop medications against *H. pylori*. This study aimed to evaluate synergistic effect of *Rubus crataegifolius* (RF) and *Ulmus macrocarpa* Hance (UL) against *H. pylori*. Antibacterial susceptibility of each extract either separately or in combination was studied against two *H. pylori* standard strains and 11 clinical isolates using agar dilution method. The effect of the extracts on *H. pylori* inoculated Balb/c mice model was also studied using single dosing (100 mg/kg each) approach. The MIC_50_ of RF and UL were more than 100 and 200 µg/ml, respectively, against the tested strains. However, simultaneous treatment with RF and UL at 75 and 50 µg/ml, respectively, showed decreased viable cell number, MIC_70_, and at 75 µg/ml each showed synergic effect with MIC_90_. On *H. pylori* inoculated Balb/c mice model, RF and UL separately (100 mg/kg each) showed moderate anti-*H. pylori* effect, while simultaneous treatment of RF and UL with same dose showed significant synergistic anti-gastric effects in stomach. The results showed a significant synergistic effect of plants extract against *H. pylori* infection and eventually gastric mucosal damage. Our finding could be considered a valuable support in the treatment of *H. pylori* induced gastritis and may contribute to the development of new and safe combined herbal product as anti-*H. pylori* regimens.

## Introduction

*Helicobacter pylori* is a pathogenic bacterium that can persist in the stomach of an infected person for their entire life. Approximately 50% of the earth's population is infected with *Helicobacter pylori*, which has been implicated in the etiology of chronic gastritis and peptic ulcer, both in adults and children (Liou et al., 2016a). The WHO has declared *H. pylori* as a carcinogen, which causes gastric cancer, the third most common cause of cancer related mortality worldwide (GBD 2013 Mortality and Causes of Death Collaborators, 2015). Several drug treatments have been developed to be efficacious in *H. pylori* infections. Triple therapy consisting of clarithromycin given for 7 to 14 days in one of the most commonly used regimens in the first line therapy (Malfertheiner et al., 2012). However, the eradication rate of standard first line therapy has fallen below 80% in many Asian countries, including China, India, and Korea (Liou et al., 2015; Liou et al., 2016b). It is noteworthy that metronidazole resistant rate was higher than 60% in Asian counties including China and India (Liou et al., 2015). In the face of the declining eradication rate of standard triple therapy, several alternative strategies have been proposed to increase the first line treatment (Rimbara et al., 2011; Gisbert and Calvet, 2012; Venerito et al., 2013). Despite advances in antimicrobial therapy, there is still no ideal treatment and indications due to increasing resistance, side effects, and falling eradication rates. Hence, considerable interest has focused on alternative/adjuvant approaches for the eradication of *H. pylori*. Some of them including herbal treatment, novel antibiotics, or classical ones from natural product in different combinations, using probiotics, etc. (Brown and Jiang, 2013; Takeuchi et al., 2014; Goderska et al., 2018).

In this work, we report in vitro anti-H. pylori activity of two popular Korean medicinal plants Rubus crataegifolius Bunge (RF, Family: Rosaceae) and Ulmus macrocarpa Hance (UL, Family:Ulmaceae) either separately or in combination and *in vivo* anti-gastritic effect of the extracts against *H. pylori* infected animal model.

## Materials and Methods

### Reagents

Dimethyl sulfoxide (DMSO), ethanol, formalin, HCl, amoxicillin, clarithromycin, omeprazole, cimetidine, ellagic acid and catechin glycoside were purchased from Sigma (Sigma Aldrich Inc., MO, USA). Fetal bovine serum (FBS) and trypsin-EDTA were obtained from GIBCO (Invitrogen INC., NY. USA). Brucella agar medium was purchased from Becton and Dickinson Company (BD). All other reagents were pharmaceutical or analytical grade.

### Plant Materials and Preparation of Extracts

The unripened fruit of RF and the stem bark of UL were purchased from Kyung Dong Medicinal Herb market at Seoul, Korea. The species of plants were verified by International Biological Material Research Center at KRIBB (Korea Research Institute of Bioscience and Biotechnology). Access number for RF Bunge (KRIBB 0001387) and *Ulmus macrocarpa* Hance (KRIBB 0002361) are kept in the herbarium of KRIBB. The experimental extracts of RF and UL were prepared using extraction, concentration and spray drying in Sam Woo-Dayeon company (Kum San, Chung Nam province, Korea). In brief, the dried fruits of RF and stem bark of UL (1 kg each) were pulverized separately using electric blender and extracted two times at 60°C for 6 hours in extraction COD water bath (JSEB-62T). Fifty percent ethanol and 100% H_2_O were used as extraction solvent (each 5 L) for RF and UL, respectively. The extracted solution was then filtered (50 mesh), concentrated with an evaporator under vacuum, spray dried, and stored at −20° C until further use. The extract yields of RF and UL were 28% and 15%, respectively. For high performance liquid chromatography (HPLC) analysis and *in vitro* assay 20% DMSO was used whereas for *in vivo* assay 5% tween 80 in H_2_O was used to solubilize the extracts.

### HPLC Analysis for Standardization of RF and UL

The quantitative analysis of the main components used as standard in *R. crataegifolius* and *U. macrocarpa* were performed using a HPLC analysis system (Agilent Technologies 1260 infinity) equipped with auto sampler (G1329B) and UV (G1316A) detector. Chromatographic separation was achieved at 35°C on Agilent reversed-phase C-18 (4.6 × 150 mm, 5 µm) column. Mobile phase A (0.1% aqueous TFA) and B (acetonitrile) with flow rate 0.8 ml/min and injection volume of 10 μl were used in analysis. The wavelength used for detection was 254 nm for ellagic acid in *R. crataegifolius* and 280 nm for catechin-7-O-β-D-apiofuranoside in *U. macrocarpa.* The quantity of ellagic acid and catechin-7-O-β-D-apiofuranoside in samples were measured from standard calibration curve (concentration vs area curve) of ellagic acid and catechin-7-O-β-D-apiofuranoside using method described by He et al., 2013.

### *Helicobacter pylori* Strains and Growth Condition

Two reference strains (ATCC-43504, SS1) obtained from American Type Tissue Culture collection (ATCC, Ritzville, ND, USA) and 11 clinically isolated strains from the Department of Microbiology, Gyeongsang National University, Korea were used for antibacterial assay. *H. pylori* (1 × 10^8^ CFU, equivalent to 1 McFarland turbidity standard unit) was seeded in brucella media containing 10% defibrinated FBS, and incubated for 24 hours at 37°C (85% N_2_, 10% CO_2_, 5% O_2_). After 3 days of incubation, number of colonies was counted. Amoxicillin was used as positive control.

### Minimal Inhibitory Concentration (MIC) Test

The minimal inhibitory concentrations were determined using the agar dilution procedure (Gisbert and Calvet, 2012) according to the guidelines described by the National Committee for Clinical Laboratory Standards (KFDA, Korea). Twenty mg of plants extract were dissolved in 1 ml of 20% DMSO and stored at −20°C. Final concentrations consisting 0, 10, 50, 75, 100, 150, and 200 µg/ml plants extract were prepared using brucella media containing 10% FBS in the Petri plates. For MIC test, each *H. pylori* strain was inoculated onto 10% FBS containing agar plates and incubated at 37°C (85% N_2_, 10% CO_2_, 5% O_2_) for 24 hours. An inoculum of each isolate of *H. pylori* strain was prepared by suspending cultured bacteria in brucella broth media to get a final inoculum concentration of 1 × 10^6^ CFU/spot. The plates were incubated at 37°C (85% N_2_, 10% CO_2_, 5% O_2_) for 3 days and the results were expressed as MIC_30_ (concentration at which inhibition of 30% of colonization occurred) or MIC_90_ values (concentration at which inhibition of 90% or more colonization).

### Animals

Male Balb/c mice, weighing 30–40 g, were purchased from Orient Bio Animal Laboratories, Kyunggi-do, Korea, and were acclimatized to standard laboratory conditions (24 ± 2°C, 45 ± 5% humidity and 12 hour light/dark cycle) for 7 days. All the procedures were performed in compliance with the guiding principles in the care of Animals and the Animal Welfare Committee of Korea Research Institute of Bioscience and Biotechnology (KRIBB, Approval No: KRIBB-AEC-14098).

### Effect of Plants Extract on *H. pylori* Induced Gastritis In Balb/c Mice

Four weeks old mice were fasted for 24 hours and then *H. pylori* culture was orally inoculated by gavage (0.5 ml, 2 × 10^8^ CFU/animal, n = 10 for each group). After inoculation, each animal was kept without food and drink for 4 hours and then given a basal diet. After 4 weeks, experimental group were received either single dose of RF and UL (100 mg/kg BW/day) or combined dose of RF and UL (100 mg/kg BW/day, 1:1 ratio) until the end of the experimental periods. The animals were monitored daily for their general health and their body weight were measured once in a week. Eight weeks after the inoculation of *H. pylori*, all animals were sacrificed under the ether anaesthesia, stomach were resected, opened along the greater curvature, and washed twice with saline. Then, gastric lesions (edema and hemorrhage) using macroscope were observed followed by measurement of the wet weight of the whole stomach including the forestomach and glandular stomach. Half of the glandular mucosa was scraped for detection of colonizing *H. pylori*, and the residual part was formalin-fixed and embedded in paraffin for histological observation. Pathological diagnosis of gastritis was made according to the criteria described previously (Takeuchi et al., 2014). A microscopic score, varying from 0 to 7, was used as a measure of the level of gastritis. To detect *H. pylori* colonization, scraped mucosa samples were homogenized, inoculated onto segregating agar plates for *H. pylori* and incubated at 37°C under microaerobic conditions. After 5 days, the colonies were counted to determine the level of *H. pylori* colonization for each stomach.

## Statistical Analysis

All experiments were performed three times and data were expressed as means ± SD. Multiple comparisons for two groups were done by independent sample T-test. *P*-values below 0.05 were considered as statistically significant. Analysis was performed using SPSS 18.0 (SPSS, Chicago, IL, United States).

## Results

### HPLC Chromatograms for Standardization of RF and UL

**Figure 1:**
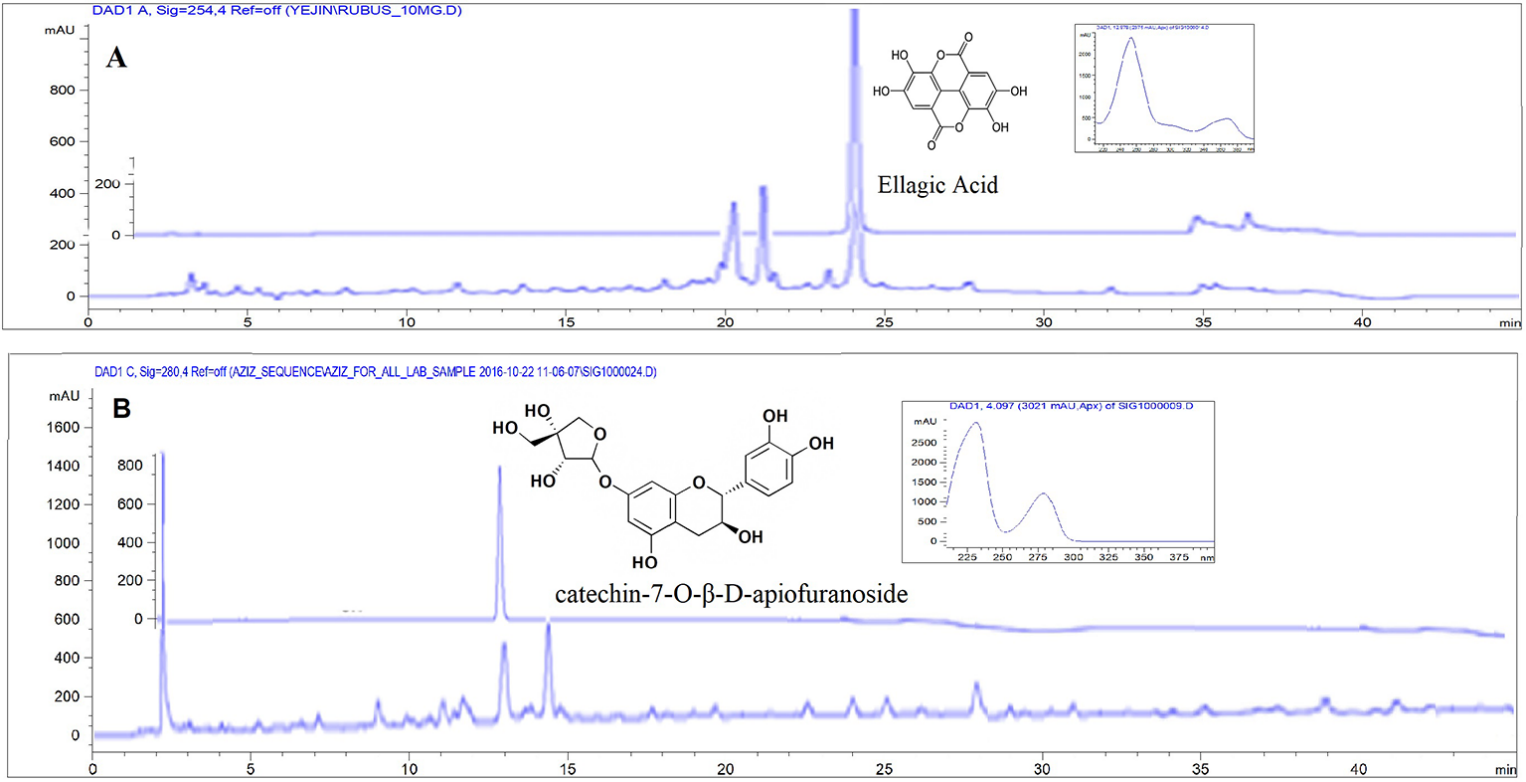
HPLC-DAD chromatogram for standardization of sample. **(A)** *Rubus crataegifolius* extracts and standard ellagic acid (overlaid) at 254 nm, **(B)** *Ulmus macrocarpa* extracts and standard catechin-7-O-β-D-apiofuranoside (overlaid) at 280 nm.

As described in *Materials and Methods*, the RF and UL used in the experiment were standardized by determining the major components using HPLC chromatography. *Rubus crataegifolius* and *Ulmus macrocarpa* samples were analyzed at 254 nm for ellagic acid and 280 nm for catechin-7-O-β-D-apiofuranoside, respectively. The Chromatogram is shown in **Figure 1**. The contents of ellagic acid in RF and catechin-7-O-ß-D-apiofuranoside in UL calculated for standardization were 14.2 and 30.5 mg/g dry extract, respectively (He et al., 2013).

### Susceptibility Study of *H. pylori* Strains

A total of 11 clinically isolated *H. pylori* strains originated from gastric ulcer of gastritis patients collected from Kyung Sang University, Korea and two reference strains obtained from ATCC were characterized for their susceptibility to amoxicillin, clarithromycin and omeprazole. The antimicrobial susceptibility results of the strains are presented in **Table 1**. All 13 strains showed to be sensitive to the tested drugs.

**Table 1:** Susceptibility test of *H. pylori* clinical and reference strains to amoxicillin, clarithromycin and omeprazole.

No.	Strain^a^	Origin	Susceptibility			
			Amoxicillin (0.5 µg/ml)	Clarithromycin (1 µg/ml)	Omeprazole (150 µg/ml)	Amo+Cla+Omp^b^
1	26695	GU	S	S	S	S
2	92-157	GU	S	S	S	S
3	92-354-4	GU	S	S	S	S
4	95-113	GU	S	S	S	S
5	95-307	GU	S	S	S	S
6	94-45	GU	S	S	S	S
7	92-20	GA	S	S	S	S
8	92-30	GA	S	S	S	S
9	92-33-1	GA	S	S	S	S
10	92-82-1	GA	S	S	S	S
11	F444-12-1	GA	S	S	S	S
12	ATCC 43504	GU	S	S	S	S
13	SS1	GU	S	S	S	S

^a^Strain 1–11 were clinical isolates from Korea and 12–13 were reference strains from ATCC; ^b^Combination of three drugs at a concentration of 0.5, 1 and 150 µg/ml, respectively; GU, gastric ulcer; GA, gastritis; S: sensitive.

### Synergistic Anti-*H. pylori* Activity of RF and UL Extracts

**Figure 2:**
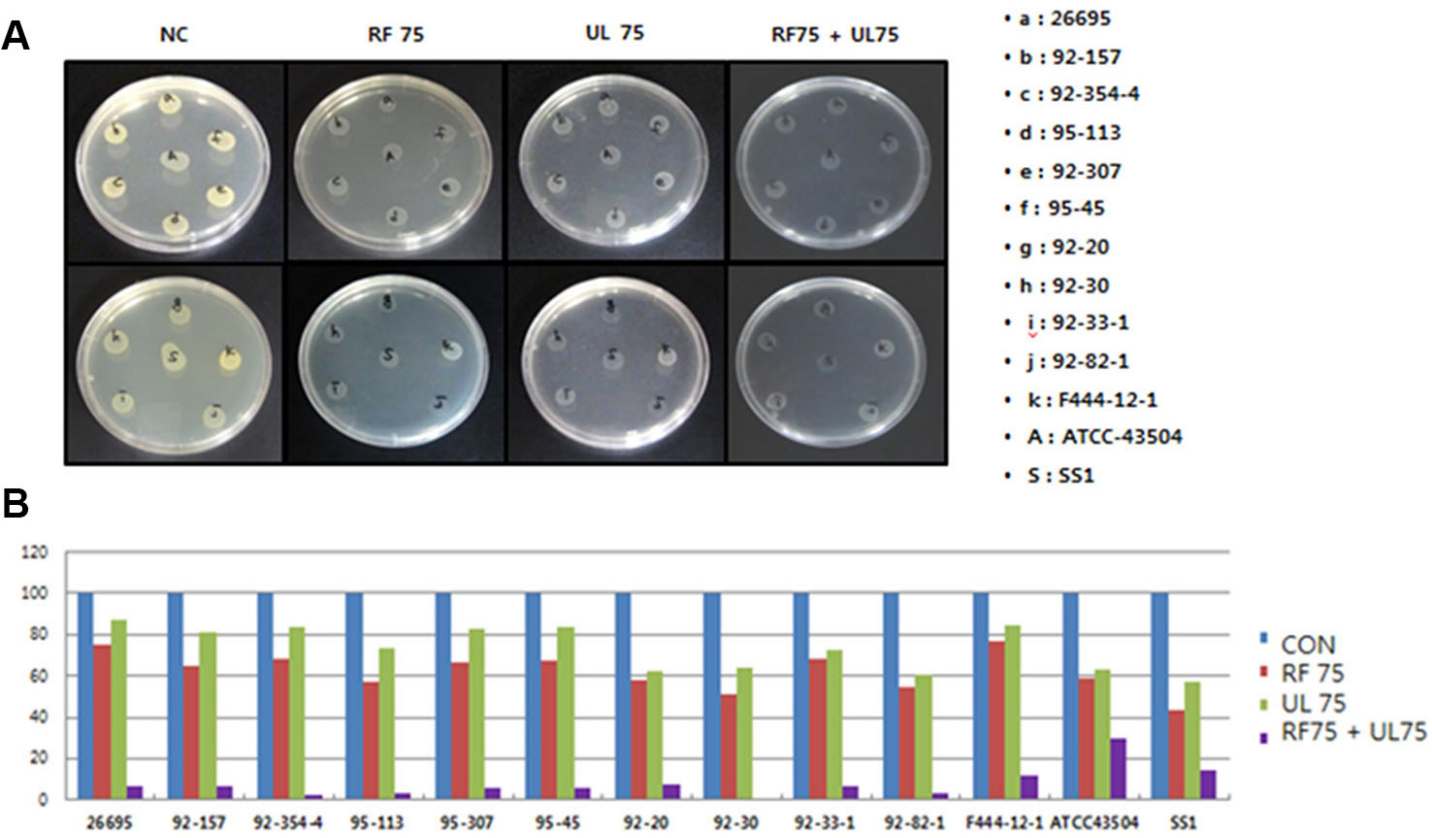
Synergistic effects of plant extracts on the colonization of *H. pylori.* **(A)** Effects of plant extracts (*Rubus crataegifolius*, *Ulmus macrocarpa*) on the colonization of 13 (represented as alphabet) *H. pylori* strains determined by agar dilution method, **(B)** Percentage of inhibition of colonization of each extract represented as graph. RF combined with UL at 75 µg/ml each showed complete inhibition of *H. pylori* growth (MIC_90_) on complex treated agar plates. CON indicated normal control and 75 indicated 75 µg/ml.

From the *in vitro* experiment using agar dilution method it was found that RF extract completely inhibited the colonization of *H. pylori* at 150 µg/ml (**Figure 2**) whereas UL showed MIC_50_ at 200 µg/ml concentrations as shown in **Table 2** and **Figure 2**. UL extract did not show any inhibition at 150 µg/ml. As shown in **Table 2**, RF or UL did not show any anti-*H. pylori* effect, at 50 or 75 µg/ml, while RF in combination with UL at 75 and 50 µg/ml, respectively, showed strong synergistic effect, MIC_70_. Combination of RF and UL at 75 µg/ml each showed complete inhibition of *H. pylori* colonization (**Figure 2**). All 11 clinically isolated *H. pylori* strains and two reference strains were tested for the synergistic effect of RF and UL. The antibiotic complex OAC (amoxicillin, clarithromycin, omeprazole) showed similar inhibitory effect on *H. pylori* colony formation (**Table 2**).

**Table 2:** Comparison of the effects of RF, UL, RF combined UL, and standard drugs on the colonization of *H. pylori*.

Sample^a^	Dose (µg/ml)	Colonization^b^	Sample^a^	Dose (µg/ml)	Colonization^b^
Control^c^	–	+++++	Catechin	50	+++++
RF	50	+++++		75	+++
	75	+++++		100	+++
	100	+++		150	+
	150	–		200	–
	200	–	Amo	50	–
UL	50	+++++		75	–
	75	+++++		100	–
	100	++++		150	–
	150	++++		200	–
	200	+++	Amo	0.5	–
RF+UL	75+50	++	Cla	1	–
	75+75	−	Omp	150	–
Ellagic acid	50	−	Amo+Cla+Omp	0.5+1+150	–
	75	−			
	100	−			
	150	−			

^a^RF, Rubus coreanus; UL, Ulmus macrocarpa; Amo, Amoxicillin; Cla, Clarithromycin, and Omp, Omeprazole; ^b^Symbol +++++, ++++, +++, ++, + and − represents MIC30 (or less), MIC40, MIC50-60, MIC70, MIC80, and MIC90 or more, respectively; ^c^Solvent only (20% DMSO in H2O).

### Suppression of H. pylori-Induced Gastritis by RF, UL, and RF Combined UL Extracts in Balb/c Mice Models

The results on the effect of plant extracts on *H. pylori* infected Balb/c mice are shown in **Table 3**. Regarding the average number of the viable bacteria, the control groups (control 2 and 3) which were infected by *H. pylori* showed 1 × 10^4^ CFU/stomach and 1 × 10^7^ CFU/stomach while positive control group (OAC treated) showed 8.2 × 10^2^ CFU/stomach. The treatment of RF and UL showed 7.5 × 10^3^ and 2.0 × 10^4^ CFU/stomach, respectively, while RF combined UL showed a significant decrement of viable *H. pylori* (1.4 × 10^3^ CFU/stomach).

**Table 3:** Effects of plant extracts on *Helicobacter pylori* infected Balb/c mice.

	Experimental group	Conditions	Log10CFU/stomach
Negative control 1	–	–	–
Negative control 2	*H. pylori* infection-4 wks	2 × 10^8^ CFU	1.0 × 10^4^
Negative control 3	*H. pylori* infection-8 wks	2 × 10^8^ CFU	7.7 × 10^7^
Positive control	OAC^a^	138+30+30 mg/kg	8.2 **×** 10^2^
Sample 1	*Rubus crataegifolius* (RL)	100 mg/kg	7.5 **×** 10^3^
Sample 2	*Ulmus macrocarpa* (UL)	100 mg/kg	2.0 **×** 10^4^
Sample 3	RL combined UL	100 mg/kg (1:1)	1.4 **×** 10^3^

^a^OAC, Omeprazole, Amoxicillin, Clarithomycin.

**Figure 3:**
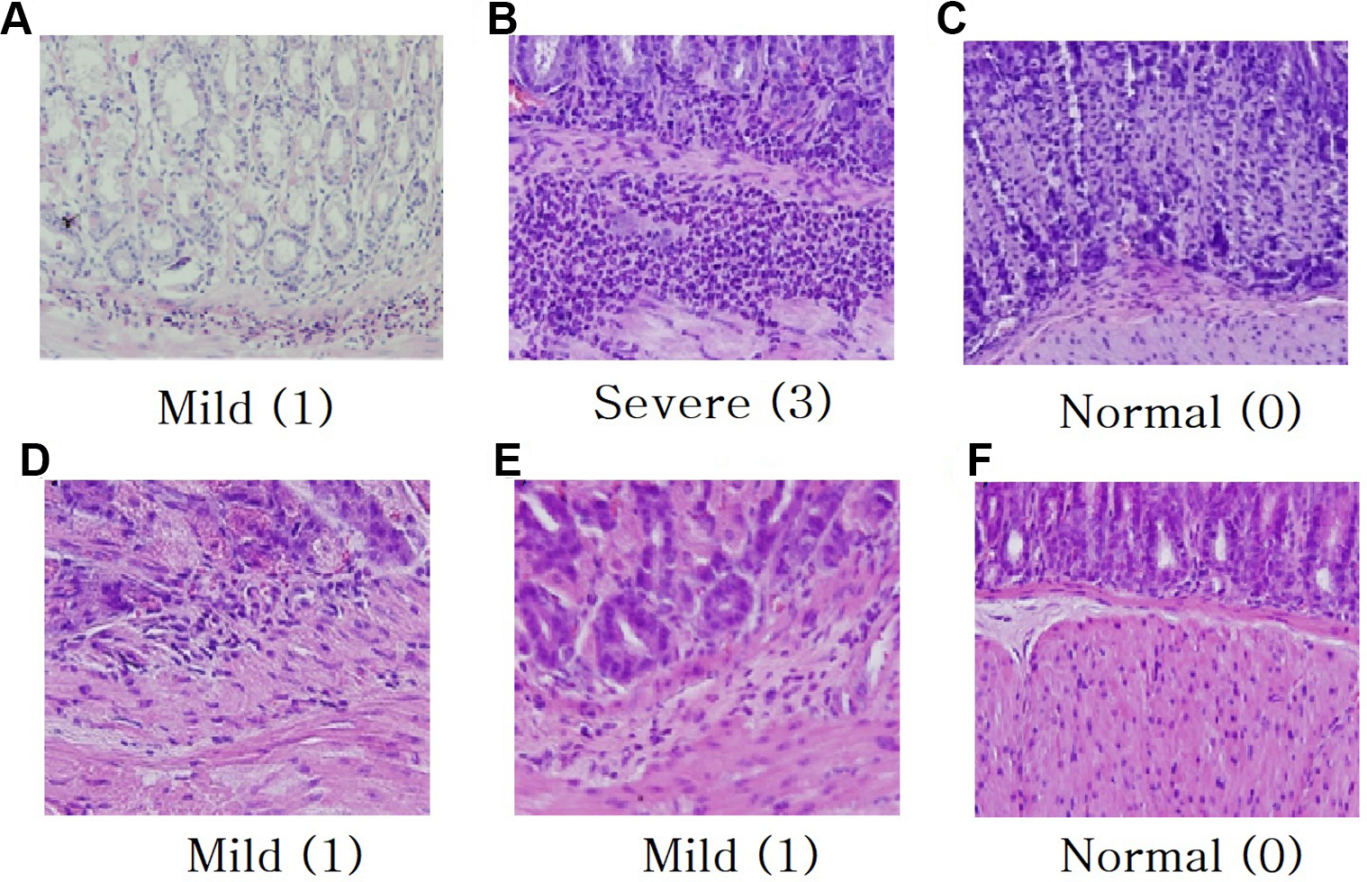
Histological analysis for H & E staining (magnification × 40). **(A)** *H. pylori* infected control mouse 4 weeks, **(B)** *H. pylori* infected control mouse 8 weeks, **(C)** Treatment with triple therapy (OAC), **(D)** Treatment with *R. crataegifolius*, **(E)** Treatment with *U. macrocarpa*, **(F)** Treatment with *R. crataegifolius* combined *U. macrocarpa*. Each plant extract suppresses immune cells against inflammation in mice stomach. Histological scoring of inflammation: normal (0), mild (1), moderate (2), and severe (3).

Histological analysis (H & E staining) is represented in **Figure 3**. Microscopic erosions with infiltration featuring many polymorphonuclear leukocyte and lymphocytes were observed in *H. pylori* infected control mouse (**Figures 3A, B**). Gastric changes were severe in the pyloric region, but moderate in fundic region. The average microscopic score for gastritis of the *H. pylori* inoculated control animal was 3.0 (**Figure 3B**). Though *H. pylori* induced gastritis showed increased immune cells in mice stomach (**Figures 3A, B**), each plants extract (**Figures 3D**–**F**) and triple therapy group (**Figure 3C**) showed suppressed immune cells against inflammation in mice stomach. The average stomach weight of control Balb/c mice inoculated with *H. pylori* was approximately 1.5-fold of that for animals without inoculation (data not shown).

## Discussion

*Rubus crataegifolius* Bunge, commonly known as “red raspberry” is used as a traditional oriental medicine in Korea. Unripened *R. crataegifolius* is extensively used in combination with other herbal preparations in food, beverages and in Korean folk medicine for its management of impotence, inflammatory, hepatotoxicity, enuresis, and allergic disease (Cha et al., 2001; Oh et al., 2007; Yang et al., 2008). The plant is also shown to possess antibacterial (Jeon et al., 2012) as well as antiulcer (Kim et al., 2011) activity. The main constituents of RF are polyphenols including sanguine, coreanoside-F1, niga-ichigoside, gallic acid, and ellagic acid (Oh et al., 2007; Kim et al., 2011; Jeon et al., 2012; Lim et al., 2012). Among these, antioxidant phenolic compound ellagic acid possesses strong chemoprotective and anti-*Helicobacter* properties (Chung, 1998). In our present study, we also found strong anti *H. pylori* effect of the plant probably due to the presence of ellagic acid at a concentration of 14.2 mg/g of dry extract. Further studies need to be done for determination whether ellagic acid is the main component which shows anti-*H. pylori* effect in RF extract.

Elm tree (UL), *Ulmus macrocarpa* Hance is a wide spread deciduous tree in Korea. The stem and root bark of this plant have long been used in oriental medicine to treat inflammation, edema, and mastitis (Yun et al., 2008; Rimbara et al., 2011; Kim et al., 2012). The plant was [reported to possess anti-gastritis, chemopreventive, and anti-*Helicobacter* activity (Yun et al., 2008; Kim et al., 2012; Hosny et al., 2014). The reported main constituents of the plant are polyphenols (Hosny et al., 2014) and it was reported that catechin showed strong anti-*Helicobacter* activity (Mabe et al., 1999). In our study, we also found moderate anti *H. pylori* effect of the plant probably due to the presence of catechin at a concentration of 30.5 mg/g of dry extract.

The significant synergistic anti-*H. pylori* and anti-gastritis effect of *R. crataegifolius* and *U. macrocarpa* is reported for the first time. The new findings are in line with the previously reported data for both plants that might encourage researchers for novel therapy schemes including phytotherapy as alternative approaches to cure *H. pylori* or as novel alimentary regimens as a more plant-based diet with an intake of compounds having chemoprotective and chemopreventive effects. Further clinical follow-up studies will be necessary to investigate the effect of active plant extracts when combined with antimicrobial agents commonly used for *H. pylori* eradications.

## Conclusion

In conclusion, the synergistic effect of two plants, RF and *Ulmus macrocarpa* against *H. pylori* infection and gastric mucosal damage is demonstrated. The results presented in this report suggest that protective synergistic effect of two well-known plants could be considered as a valuable support in the treatment of *H. pylori* induced gastritis and may contribute to the development of new and safe agents of inclusion in anti-*H. pylori* regimens.

## Data Availability Statement

All datasets generated for this study are included in the article.

## Ethics Statement

The animal study was reviewed and approved by the Care of Animals and the Animal Welfare Committee, Korea Research Institute of Bioscience and Biotechnology (KRIBB).

## Author Contributions

JP: Experimental design for *Helicobacter* and MIC test using RF and UL. JC: Purchasing and processing of RF and UL. JK and HK: MIC test and gastritis experiment. YJ: Histological analysis, H & E staining. MR: HPLC analysis and *in vitro Helicobacter* activity test. YL: Conception and design, participated in general coordination of the study, writing and revising the manuscript.

## Funding

The research was supported by Korea Ministry of SMEs (Small and Medium Enterprise) and Startups with grant number #2410060 and Lee's Biotech Co., KRIBB, Korea. Lee's Biotech provided funding/resources for analysis including extraction and HPLC analysis.

## Conflict of Interest

Authors JC and MR are employed by Lees Biotech Co. Authors HK and YJ are employed by Kolmar BNH Co.

The remaining authors declare that the research was conducted in the absence of any commercial or financial relationships that could be construed as a potential conflict of interest.
